# Neuronal Panx1 drives peripheral sensitization in experimental plantar inflammatory pain

**DOI:** 10.1186/s40779-024-00525-8

**Published:** 2024-04-29

**Authors:** Qu Xing, Antonio Cibelli, Greta Luyuan Yang, Preeti Dohare, Qing-Hua Li, Eliana Scemes, Fang-Xia Guan, David C. Spray

**Affiliations:** 1 School of Life Sciences, Zhengzhou University, Zhengzhou, 450001 China; 2 Dominick P. Purpura Department of Neuroscience, Albert Einstein College of Medicine, Bronx, NY 10461 USA; 3 Department of Biosciences, Biotechnologies and Biopharmaceutics, University of Bari Aldo Moro, Bari, 70125 Italy; 4 Department of Molecular Biology and Biochemistry, Wesleyan University, Middletown, CT 06459 USA; 5 Department of Neuroscience and Experimental Therapeutics, Albany Medical College, Albany, NY 12208 USA; 6 Department of Anatomy and Cell Biology, New York Medical College, Valhalla, NY 10595 USA; 7 Institute of Neuroscience, Zhengzhou University, Zhengzhou, 450001 China

**Keywords:** Panx1, Dorsal root ganglion, Satellite glial cell, Peripheral sensitization, Plantar inflammatory pain

## Abstract

**Background:**

The channel-forming protein Pannexin1 (Panx1) has been implicated in both human studies and animal models of chronic pain, but the underlying mechanisms remain incompletely understood.

**Methods:**

Wild-type (WT, *n =* 24), global *Panx1* KO (*n =* 24), neuron-specific *Panx1* KO (*n =* 20), and glia-specific *Panx1* KO (*n =* 20) mice were used in this study at Albert Einstein College of Medicine. The von Frey test was used to quantify pain sensitivity in these mice following complete Freund’s adjuvant (CFA) injection (7, 14, and 21 d). The qRT-PCR was employed to measure mRNA levels of *Panx1*, *Panx2*, *Panx3*, *Cx43*, *Calhm1*, and *β-catenin*. Laser scanning confocal microscopy imaging, Sholl analysis, and electrophysiology were utilized to evaluate the impact of *Panx1* on neuronal excitability and morphology in Neuro2a and dorsal root ganglion neurons (DRGNs) in which Panx1 expression or function was manipulated. Ethidium bromide (EtBr) dye uptake assay and calcium imaging were employed to investigate the role of Panx1 in adenosine triphosphate (ATP) sensitivity. β-galactosidase (β-gal) staining was applied to determine the relative cellular expression levels of Panx1 in trigeminal ganglia (TG) and DRG of transgenic mice.

**Results:**

Global or neuron-specific *Panx1* deletion markedly decreased pain thresholds after CFA stimuli (7, 14, and 21 d; *P* < 0.01 vs. WT group), indicating that Panx1 was positively correlated with pain sensitivity. In Neuro2a, global *Panx1* deletion dramatically reduced neurite extension and inward currents compared to the WT group (*P* < 0.05), revealing that Panx1 enhanced neurogenesis and excitability. Similarly, global *Panx1* deletion significantly suppressed Wnt/β-catenin dependent DRG neurogenesis following 5 d of nerve growth factor (NGF) treatment (*P* < 0.01 vs. WT group). Moreover, Panx1 channels enhanced DRG neuron response to ATP after CFA injection (*P* < 0.01 vs. *Panx1* KO group). Furthermore, ATP release increased Ca^2+^ responses in DRGNs and satellite glial cells surrounding them following 7 d of CFA treatment (*P* < 0.01 vs. *Panx1* KO group), suggesting that Panx1 in glia also impacts exaggerated neuronal excitability. Interestingly, neuron-specific *Panx1* deletion was found to markedly reduce differentiation in cultured DRGNs, as evidenced by stunted neurite outgrowth (*P* < 0.05 vs. *Panx1* KO group; *P* < 0.01 vs. WT group or GFAP-Cre group), blunted activation of Wnt/β-catenin signaling (*P* < 0.01 vs. WT, *Panx1* KO and GFAP-Cre groups), and diminished cell excitability (*P* < 0.01 vs. GFAP-Cre group) and response to ATP stimulation (*P* < 0.01 vs. WT group). Analysis of β-gal staining showed that cellular expression levels of Panx1 in neurons are significantly higher (2.5-fold increase) in the DRG than in the TG.

**Conclusions:**

The present study revealed that neuronal Panx1 is a prominent driver of peripheral sensitivity in the setting of inflammatory pain through cell-autonomous effects on neuronal excitability. This hyperexcitability dependence on neuronal Panx1 contrasts with inflammatory orofacial pain, where similar studies revealed a prominent role for glial Panx1. The apparent differences in Panx1 expression in neuronal and non-neuronal TG and DRG cells are likely responsible for the distinct impact of these cell types in the two pain models.

**Supplementary Information:**

The online version contains supplementary material available at 10.1186/s40779-024-00525-8.

## Background

Pain sensitivity is amplified and becomes persistent through both peripheral and central sensitization mechanisms [1–3]. Chronic inflammatory pain is complex, involving not only tissue damage but also local release of inflammatory signals that aggravate and prolong allodynia. Traditional pharmacological approaches [opioids, cannabinoids, and non-steroidal anti-inflammatory drugs (NSAIDs)] have made great progress in reducing inflammatory pain, but they present potential risks in clinical practice [4, 5]. For instance, long-term analgesic usage may elevate the risk of kidney and cardiovascular disease [6, 7], and pain-relieving opioid and cannabinoid drugs can be addictive [8–10]. Thus, it is critical to explore novel drug targets for the development of inflammatory pain therapies.

Peripheral pain sensitization consists of a reduced threshold for painful sensitivity (allodynia /hyperalgesia) [11], and the dorsal root ganglion (DRG) thus offers a target for neuromodulation in the transmission of pain signals to the dorsal horn of the spinal cord in chronic inflammatory pain [12–14]. Inflammatory injury intensifies interactions between sensory neurons and satellite glial cells (SGCs) surrounding them, contributing to persistent hyperexcitability through pathophysiological changes in DRG neurons (DRGNs) [15, 16]. These changes offer potential novel therapeutic targets for DRG-based pain modulation in inflammatory pain.

Pannexin1 (Panx1) has been shown to be involved in chronic pain and could thus be a target following inflammatory pain insult [17–20]. As a non-selective and large–conductance channel, Panx1 provides a conduit for the release of adenosine triphosphate (ATP) [21–24], which has long been known to enhance neuron excitability and to be activated by both mechanical and chemical signals [25–31]. Critically, *Panx1* is expressed in both neurons and SGCs in sensory ganglia, and its expression increases in the ganglia in chronic inflammatory pain models [17, 20].

In neuropathic pain models [32, 33], Panx1 blockade by gene deletion or small molecule inhibitors has been shown to reduce hypersensitivity. We have previously demonstrated that both glial and neuronal Panx1 in the trigeminal ganglia (TG) contribute to orofacial pain hypersensitivity in an inflammatory mouse model [18, 34, 35], and another study reported that Panx1 channel in immune cells contributes to inflammatory pain originating in the DRG [36]. However, the underlying mechanism by which Panx1 contributes to inflammatory pain is largely unresolved.

In this study, we aimed to investigate how Panx1 controls peripheral pain sensitization after inflammatory stimuli. *Panx1* levels in the DRG were found to be increased in a mouse model of chronic inflammation-induced pain, and in mice lacking *Panx1*, either globally or in neurons, pain hypersensitivity was much attenuated. SGC deletion of *Panx1*, previously found to appreciably reduce inflammatory orofacial pain, was less effective than neuronal deletion in the hindlimb inflammatory pain model, consistent with the lower SGC than neuronal *Panx1* expression we detected in DRG compared to TG. Deletion of *Panx1* in DRG and Neuro2a neurons resulted in numerous deficits in morphology and function, including much-reduced neurite extension, decreased excitability, and impaired ATP release. These studies showing major impact of Panx1 on peripheral inflammatory pain may provide a novel therapeutic strategy in future clinical trials and drug development.

## Methods

### Animal

The Institutional Animal Care and Use Committee (IACUC) of Einstein approved all animal experiments (NO. IACUCs20151101), assuring compliance with ethical and legal requirements for animal research.

The 8–12-week-old mice (*n =* 48) were used in the course of the study: wild-type (WT, *n =* 24), global *Panx1* knockout (KO) (*Panx1*^−/−^, *n =* 24), neuron-specific *Panx1* KO (NFH-Cre: *Panx1*^f/f^, abbreviated NFH-Cre, *n =* 20), and glia-specific *Panx1* KO (GFAP-Cre: *Panx1*^f/f^, abbreviated GFAP-Cre, *n =* 20). Global *Panx1* KO and neuron/glia-specific *Panx1* KO mice were generated in the C57BL/6 background at the IACUC-approved animal facility of Albert Einstein College of Medicine, as previously reported [21]. Briefly, homozygous global *Panx1* KO was generated from hemizygous *Panx1*^tm1a(KOMP)Wtsi^ mice (KOMP, Davis, CA, USA). *Panx1*^f/f^ mice were obtained by crossing *Panx1*^tm1a (KOMP)Wtsi^ with flippase deleter mice (B6.ACTFLPe/J), allowing for neuron/glia-specific *Panx1* knockout mice strain generation following crossing with cell-type specific Cre mice (Jackson Laboratory, Bar Harbor, ME, USA). Glia-specific *Panx1* KO mice were generated by crossing mGFAP-Cre [B6.Cg-Tg (Gfap-cre) 73.12 Mvs/J] and *Panx1*^f/f^ mice. Neuron-specific *Panx1* KO mice were established by crossing mNFH-Cre [Tg (Nefh-cre) 12 Kul/J] and *Panx1*^f/f^ mice.

### Plantar inflammatory pain model

The plantar inflammatory pain was induced by complete Freund’s adjuvant (CFA, #F5881, Sigma-Aldrich, Germany), following the previously established procedure [18]. For that, 20 µl of CFA solution (1 mg/ml, prepared by mixing 1:1 saline to CFA) was injected subcutaneously into the plantar portions of the right hind paw under anesthesia (isoflurane).

### Pain sensitivity analysis

Wild-type (*n =* 11), global *Panx1* KO (*n =* 11), neuron-specific *Panx1* KO (*n =* 12), and glia-specific *Panx1* KO mice (*n =* 12) were used in the behavioral test. Mouse paws were stimulated with von Frey filaments (Stoelting Co., Dale Wood, IL, USA) to measure tactile sensitivity as previously described [17]. Briefly, the mice were placed in elevated plastic boxes with a wire mesh floor and each filament was applied ten times with 5–30 s random intervals to target the paw. Tactile thresholds (expressed in g) were measured as the calibrated bending forces of the filament that elicited a withdrawal response. Data for 50% pain thresholds were collected on 0, 7, 14, and 21 d, and represented as three-dimensional (3D) surface plots and two-dimensional (2D) heatmaps of the number of responses to 10 stimulus presentations of each intensity using the NSCC software (version 2019, NSCC, Kaysville, UT, USA).

### Cell culture

Details regarding the generation of *Panx1* CRISPR KO and overexpressing Neuro2a cell lines were previously described [37]. Neuro2a and Neuro2a CRISPR KO *Panx1* and overexpressing *Panx1* cells were cultured in DMEM (#8,121,513, Gibco, USA) supplemented with 10% fetal bovine serum (FBS; #10,099,141, Gibco, USA). To stimulate neuronal outgrowth, those Neuro2a cell lines above were differentiated by a 5 d treatment with 40 µmol/L retinoic acid (RA; #302-79-4, Merck, Germany) to specifically investigate the function of Panx1 channels.

Sensory neurons were dissociated from L5 DRG following painless death (isoflurane followed by decapitation) using collagenase/dispase digestion [38] from WT, *Panx1* mutant, and CFA-treated mice at the same time point. Then, a total of 20,000 neurons were plated in 60-mm dishes coated with poly-d-lysine (PDL; 25 µg/ml, #LPDL001, Sigma-Aldrich, Germany) and then cultured in 5 µg/ml nerve growth factor (NGF; #9061-61-4, Sigma-Aldrich, Germany) for 5 d [39].

To explore the signaling pathways underlying dendritic growth during DRG neuron culture, an agonist (20 mmol/L LiCl, #7447-41-8, Selleck Chemicals, USA) was added to activate Wnt/β-Catenin signaling by limiting β-catenin phosphorylation and degradation [40], Wnt ligand blocker (30 nmol/L IWP2, #686770-61-6, Merck, Germany) was added to prevent early Wnt ligand palmitoylation by targeting the O-acyltransferase porcupine (Porcn) [41], and β-catenin inhibitor (100 nmol/L Triptonide, #38647-11-9, Selleck Chemicals, USA) was added to block the Wnt pathway by binding to β-catenin’s C-terminal Transactivation Domain [42].

### Cell transfection

The 3 µg of enhanced green fluorescent protein (EGFP)-labelled plasmid m*Panx1*-DNA with 10 µl of transit LT1 transfection reagent (#MIR-2304, Mìrus, USA) in 250 µl of Opti-MEM (#31,985,062, GIBCO, USA) were used to re-express *Panx1* in Neuro2a *Panx1* KO cells. Cell culture medium was replaced with standard growth medium [DMEM (#8,121,513, Gibco, USA) supplemented with 10% FBS (#10,099,141, Gibco, USA)] after 24 h of transfection.

### Neurite outgrowth assessment

For quantification of neural differentiation, cells were viewed using phase-contrast microscopy (20×) with a Nikon Eclipse TE300 microscope (Nikon Co., Minato, TY, Japan). Five fields from each cell culture were randomly selected, imaged, and then measured using ImageJ (NIH, Bethesda, MD, USA) [43]. At least 30 cells in each random field of view were studied to calculate the neurite-bearing cell ratio using the following formula:

$$\varvec{Ratio\;(\%)}=(\sum \text{neurite}\_\text{bearing}/\sum \text{neuron})\times100$$

where ∑neurite_bearing is the number of neurite-bearing cells and ∑neuron is the total number of neurons. A cell was defined as neurite-bearing if it had at least one neuronal process that was longer than its soma.

### Sholl analysis

Sholl analysis was performed on 8-bit binary images of selected neurofilament-200 (NF-200)-stained DRGNs (neurite length ≥ 20 μm) using the Sholl analysis plug-in (ImageJ, NIH, Bethesda, MD, USA). Dendrite intersections were automatically evaluated at 10-µm radius intervals starting from the soma. Primary and secondary dendrite lengths were determined by measuring the distance of each radius from the soma/branch point.

### Ethidium bromide (EtBr) uptake

DRGNs were seeded in monolayers on glass-bottomed dishes (MatTek, USA) at a density of 2000 cells/cm^2^, and then cultured with DMEM medium (#8,121,513, Gibco, USA) with 10% FBS (#10,099,141, Gibco, USA) for 5 d. After rinsing twice with PBS without Ca^2+^ and Mg^2+^ (#21-031-CV, Corning, USA), DRGNs were incubated for 10 min in 5 ml of DMEM with 10 µmol/L EtBr (#E7637, Merck, Germany) and 2 mmol/L ATP (#R0441, Thermo Scientific, USA). The fluorescence intensity of EtBr dye uptake was monitored using a Nikon Eclipse TE300 microscope (Nikon Co., Minato, TY, Japan) and Spot-RT digital camera with fixed gain and exposure duration.

### Extracellular ATP release

As previously described [17], the extracellular ATP released from DRG ganglia was determined using the D-luciferin/luciferase assay (#A22066 ATP Determination Kit, Thermo Scientific, USA). Briefly, excised DRG ganglia of mice one week after CFA injection into hindlimb paw were immediately incubated in glass-bottomed dishes (MatTek, USA) containing ACSF (125 mmol/L NaCl, 3.5 mmol/L KCl, 2.0 mmol/L CaCl_2_, 2.0 mmol/L MgSO_4_, 1.25 mmol/L KH_2_PO_4_, 26 mmol/L NaHCO_3_, 10 mmol/L D-glucose, pH 7.2) at 37 °C for 40 min. Then, 500 µl of ACSF was collected and evaluated using an ATP Determination Kit according to the manufacturer’s instructions. Finally, the luminescence of ACSF at 560 nm was monitored using a TD 20/20 luminometer (Turner Designs, Sunnyvale, CA, USA). The ATP values released by the DRG were calculated based on the standard curves and normalized to the total protein amount, which was quantified using a bicinchoninic acid assay (#23,225, Thermo Scientific, USA).

### Calcium imaging

DRGNs were loaded with the ratiometric Ca^2+^ indicator Fura-2 acetoxymethyl ester (5 µmol/L; #F1221, Invitrogen, USA) at 37 °C for 30 min, rinsed, and kept in phenol-free α-MEM supplemented with 1% FBS (#10,099,141, Gibco, USA) and 20 mmol/L HEPES (#7365-45-9, Sigma-Aldrich, Germany) throughout each test. Cells were imaged on a Nikon TE 2000 microscope (20 × 0.65 NA objective; Nikon Co., Minato, TY, Japan) with MetaFluor software (Molecular Devices Co., San Jose, CA, USA) using a Hamamatsu camera and Sutter filter changer connected to the microscope via a fiber optic cable. Intracellular Ca^2+^ was measured at 2 Hz by imaging regions of interest (ROIs) corresponding to individual cell bodies with 380/340 nm dual excitation (Sutter filter changer) and Fura-2 filter cube. As described in our earlier study [44], ratio values were converted to intracellular Ca^2+^ concentrations using in vitro calibrations in MetaFluor software (version 6.1, Universal Imaging. Corp., USA). Next, 2 mmol/L ATP was added to the bathing solution after 10 s of 200 s tracing to stimulate cellular Ca^2+^ responses. ROIs on at least 10 cell bodies were used to determine Ca^2+^ responses in each test. Data were normalized to the average non-ATP treated baseline amplitudes.

### RNA extraction and qRT-PCR

RNA samples were extracted from Neuro2a cells, *Panx1* KO DRGNs, Wnt/β-catenin agonist/inhibitor-treated DRGNs, and DRG tissue using TRIzol reagent (#15,596,026, Invitrogen, USA) for qRT-PCR with corresponding primers (Additional file 1: Table S1). The cDNA was synthesized from 1 µg of RNA sample using SuperScript™ IV VILO™ Master Mix (#11,756,500, Invitrogen, USA) according to the manufacturer’s instructions.

The qRT-PCR assay was performed using Power SYBR® Green RNA-to-CT™ 1-Step Kit (#4,389,986, Thermo Scientific, USA) and QuantStudio™ 6 Flex system (Life Technologies, Carlsbad, CA, USA). Reaction mixtures were denatured at 95 °C for 10 min followed by 40 PCR cycles. Each cycle consisted of the following three steps: 94 °C for 15 s, 57 °C for 15 s, and 72 °C for 1 min. The ddCT method was employed to determine relative gene-level differences with GAPDH qPCR products used as the control.

### Electrophysiology

Whole-cell patch clamp was utilized to determine the amplitude of voltage-activated membrane currents in Neuro2a and DRG cells before and after the differentiating treatments. Cells were bathed in PBS solution containing 1 g/L glucose at pH 7.2. Patch pipettes with tip resistance of 3–6 MΩ were filled with an internal solution consisting of K-gluconate (140 mmol/L), HEPES (10 mmol/L), MgCl_2_ (1 mmol/L), EGTA (1 mmol/L), and Na_2_ATP (4 mmol/L) at pH 7.2. Membrane currents were recorded using an Axopatch-1B amplifier with a digitizer and stored in a computer for analysis using pClamp10 (Molecular Devices Co., San Jose, CA, USA). Leakage subtraction was employed in some recordings using standard protocols. Values reported for inward current amplitudes generally reflected such subtraction. Panx1 blocker probenecid (Pbcd; 1 mmol/L, #57-66-9 Sigma-Aldrich, Germany) was added during cell differentiation in Neuro2a *Panx1* KO, Neuro2a WT, Neuro2a *Panx1* OE, DRGNs WT, and *Panx1* KO DRGNs groups.

### Immunostaining

The DRGNs on PDL-coated coverslips or DRG tissue Sects. (10–15 μm) were fixed using 4% PFA solution (#158,127, Sigma-Aldrich, Germany) in phosphate-buffered saline (PBS) for 15 min, permeabilized with 0.4% Triton-X 100 (#93,443, Sigma-Aldrich, Germany) in 1× PBS for 10 min, and blocked at room temperature with PBS buffer containing 0.4% Triton-X 100 and 10% goat serum (#16,210,064, Thermo Scientific, USA). The coverslips and sections were stained with primary antibodies targeting NF200 (1:200; rabbit IgG; #N4142, Sigma-Aldrich, Germany) and glutamine synthetase (1:500; mouse IgG, #sc-74,430, Santa Cruz, USA) in the incubating solution (0.4% Triton-X 100 and 2% goat serum) for at least 24 h at 4 °C. Following three gentle rinses with 1X PBS, the samples were incubated with secondary antibodies that included donkey anti-rabbit IgG-Alexa Fluor™488 (1:1,000; #A-21,206, Invitrogen, USA) and goat anti-mouse IgG (H + L) - Alexa Fluor™ 594 (1:1,000, #A-11,032, Invitrogen, USA) at 37 °C for 2 h. After three PBS washes in the dark, the samples were mounted using the mounting medium (#H-1000-10, Vector Laboratories, USA) containing 1% 4’6-Diamidino-2-phenylindole stock (DAPI, 10 mmol/L, #D9542, Sigma-Aldrich, Germany). The coverslips and DRG tissues were photographed using a Zeiss LSM 880 confocal microscope equipped with a Plan-Apochromat 63x/1.40 NA Oil DIC objective (Carl Zeiss AG, Jena, TH, Germany).

### β-galactosidase (β-gal) Staining

β-gal staining in TG and DRG sections was performed as previously described [17]. Briefly, tissues were fixed in 4% PFA in PBS for 1 h, rinsed with PBS, washed with the washing buffer (2.0 mmol/L MgCl_2_, 0.01% deoxycholate, 0.02% NP-40, 97 mmol/L Na_2_PO_4_, pH 7.3) for 30 min, and incubated overnight at 37 °C with X-gal reaction solution [1.0 g/L X-gal (#R0941, Thermo Scientific, USA), 5 mmol/L potassium ferrocyanide (#14459-95-1, Merck, Germany), 7 mmol/L potassium ferricyanide]. After rinsing with the washing buffer, tissues were post-fixed overnight in 4% PFA and then immersed in (5, 10, 20, and 30%) sucrose solution in PBS for 24 h each at 4 °C. The tissues were then embedded in an OCT compound and immediately frozen at − 80 °C. Sections 10–15 μm in thickness were cut using a cryostat at − 20 °C. Tissues were examined with a Leica inverted DMi8 SP8 microscope. Leica LASX software was used for image acquisition (Leica Microsystems CMS GmbH, Mannheim, BW, Germany). Images were analyzed using ImageJ software (NIH, Bethesda, MD, USA). The number of stained cells (neurons or satellite glial cells) was normalized to the total number of neurons.

### Statistical analysis

Measurement data were expressed as mean ± standard error of the mean (SEM) from at least three independent replicates. Data were analyzed using SPSS (version 22, IBM Co., Armonk, NY, USA) and plotted with Prism software (version 9.5.1, GraphPad, Armonk, NY, USA). T-test was used to compare two groups except for β-gal staining, where unpaired Student’s *t*-test was utilized. One-way ANOVA was carried out for multiple comparisons. Two-way ANOVA with Bonferroni post hoc test was performed to compare the variables between different genotypes, including 50% pain threshold and *β-catenin* relative level test. All statistical details are shown in the respective figure legend. A *P*-value of ≤ 0.05 indicated statistical significance.

For behavioral studies, at least 10 mice were included in each animal group based on the power analysis recommendation [23], and tests were scheduled at their appointed time.

## Results

### Global or neuron-specific *Panx1* deletion decreases pain sensitivity

**Fig. 1:**
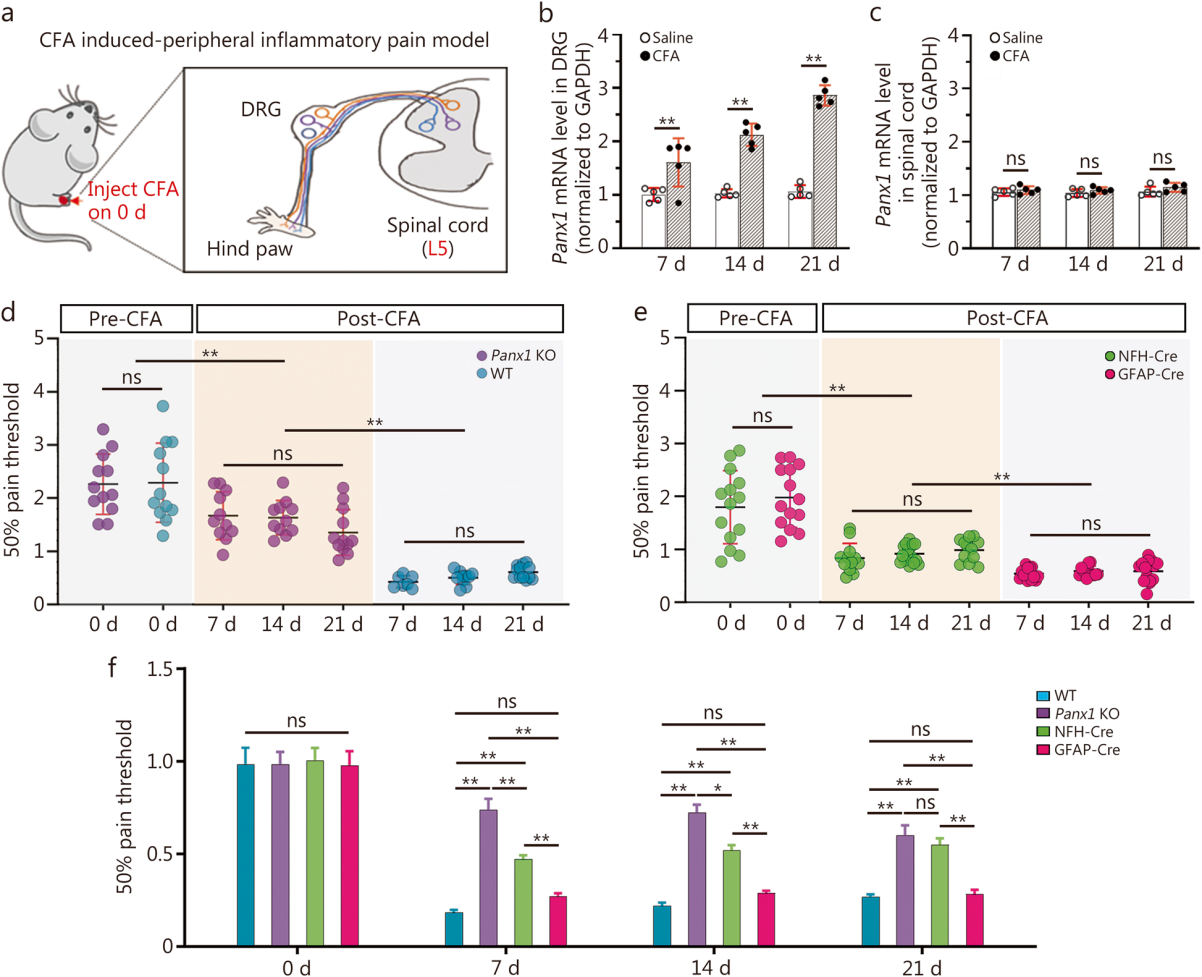
Global or neuronal *Panx1* deletion reduces allodynia while GFAP-targeted deletion does not. **a** Schematic plot of CFA induced peripheral inflammatory pain model. *Panx1* mRNA expression in DRG (**b**) and spinal cord (**c**) from WT and *Panx1* KO mice within 3 weeks after CFA inflammation. **d** Three-week dynamic 50% pain threshold of WT and *Panx1* KO mice. **e** Three-week dynamic 50% pain threshold of NFH-Cre and GFAP-Cre *Panx1* KO mice. **f** Comparison of pain sensitivity changes among those four genotypes above at each time point. For panels **b** and **c**, *n =* 3 in saline and CFA group; *n =* 11 in WT group, *n =* 11 in *Panx1* KO group, *n =* 12 in GFAP-Cre group, and *n =* 12 in NFH-Cre group. Two-way ANOVA with Bonferroni, interaction F (2, 12) = 26.07 in panel **b**, *F* (2, 12) = 0.1176 in panel **c**, *F* (7, 68) = 29.66 in panel **d**, *F* (7, 87) = 33.44 in panel **e**, and *F* (9, 170) = 5.641 in panel **f**. ns non-significant, ^*^P < 0.05, ^**^P < 0.01. CFA complete Freund’s adjuvant, DRG dorsal root ganglion, NFH-Cre neuro filament H-Cre, GFAP-Cre glial fibrillary acidic protein-Cre, Panx1 pannexin 1, Panx1 KO pannexin 1 knockout, WT wild-type

In this study, the inflammatory agent CFA was injected into the mouse hind paw (Fig. 1a) to test whether *Panx1* expression in sensory ganglia was altered during chronic pain. As shown in Fig. 1b, gene expression of *Panx1* was significantly progressively increased in L5 DRG from 7 to 21 d after CFA injection. However, there was no increase in *Panx1* mRNA in the L5 spinal cord (dorsal and ventral horns; Fig. 1c). Pain threshold values showed a more substantial and significant increase in global *Panx1* KO mice, compared to those WT mice (Fig. 1d). Normalized values of 50% pain thresholds (post-/pre-CFA) in WT were strikingly lower than in global *Panx1* deletion from 7 d till 21 d (Additional file 1: Fig. S1a). The positive response fraction to each magnitude of von Frey filament applied force at each time point is shown in pseudo-color displays (Additional file 1: Fig. S1c). As shown in the 3D convex topology of WT mice, there are fewer responses to weak stimuli and more responses to moderate and robust stimuli, compared to the planar topology in *Panx1* KO group. This difference was apparent as a narrower blue band and a more intense red region in WT in its 2D plots. For *Panx1* KO mice, the blue region was particularly pronounced on 14 d and 21 d, while the red intensity was lower at all time points. These results indicated that *Panx1* deletion substantially blocks CFA administration-induced peripheral hypersensitivity.

Interestingly, further allodynia studies showed a difference between neuronal and glial *Panx1* deletion mice. As shown in Fig. 1e, the pain sensitivity baseline (pre-CFA) was similar between those two groups. But neuron-specific *Panx1* deletion (NFH-Cre) led to a significant decrease in pain sensitivity persisting for 3 weeks after CFA injection, compared to only minor differences in the glia-specific *Panx1* deletion (GFAP-Cre) mice. Also, normalized 50% pain thresholds (post-/pre-CFA) in the GFAP-Cre group were markedly less decreased than in the NFH-Cre *Panx1* KO mice from 7 to 21 d, indicating that neuronal *Panx1* was critical for hypersensitivity attenuation (Additional file 1: Fig. S1b). The 3D plots indicated there were more numerous paw withdrawal responses in GFAP-Cre:*Panx1*^f/f^, particularly at the highest stimulus intensities and at longer times after inflammation. The 2D heatmaps of the GFAP-Cre mice showed that the extent and saturation of the red region were much higher than in NFH-Cre mice (Additional file 1: Fig. S1d). These results also confirmed that allodynia was significantly lower in the NFH-Cre group.

For pain threshold baselines, there were no significant differences among WT, *Panx1* KO, NFH-Cre, and GFAP-Cre mice before CFA injection (Additional file 1: Fig. S2). Pain sensitivity was dramatically attenuated in global *Panx1* KO/NFH-Cre mice, compared to WT and GFAP-Cre groups at all time points (Fig. 1f). The differences were not attributable to gross changes in mouse body weight, which was well maintained in all four genotypes (Additional file 1: Fig. S1e). Therefore, global/neuronal *Panx1* KO but not glial *Panx1* deletion decreased pain sensitivity in the CFA-induced inflammatory pain model.

### *Panx1* deletion decreases neurite extension and inward current in Neuro2a

**Fig. 2:**
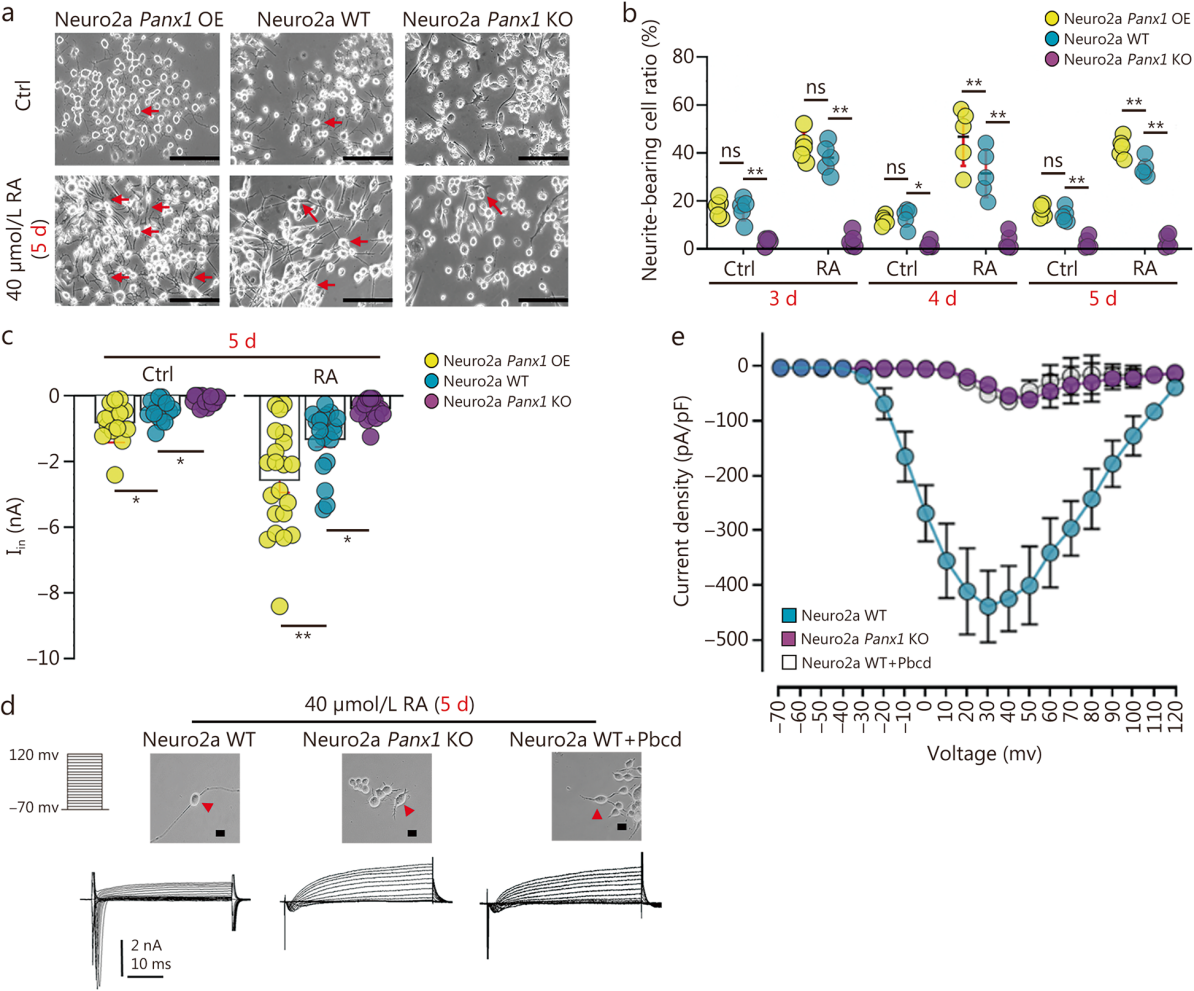
Panx1 facilitates neurite extension and promotes cell excitability in Neuro2a neuroblastoma cells. **a** Representative photomicrograph of Neuro2a WT, Neuro2a *Panx1* KO, and Neuro2a *Panx1* OE cells after 5 d of RA treatment. Arrows indicate processes of representative cells in each condition. **b** Neurite extension analysis in WT, Panx1-deleted, and Neuro2a Panx1 OE cells at d 3–5 in culture with or without 40 µmol/L RA (*n =* 5). c Quantification of peak inward currents in Neuro2a WT, Neuro2a *Panx1* KO and Neuro2a *Panx1* OE on day 5 with and without RA treatment (*n* = 15–22 cells in each group). d Representative recordings of whole-cell voltage-clamp currents in Neuro2a WT, Neuro2a *Panx1* KO, and Neuro2a WT plus Panx1 blocker 1 mmol/L Pbcd following 5 d treatment with RA. **e** Current density analysis of Neuro2a cells with 1 mmol/L Pbcd (*n =* 9–11 in each group). Two-way ANOVA with Bonferroni in **b, c**, interaction *F* (2, 24) = 15.04 in panel **b**, *F* (2, 56) = 18.62 in panel **c**; scale bar = 150 μm in panel **a** and scale bar = 20 μm in panel **d**. ns non-significant, ^*^P < 0.05, ^**^P < 0.01. Neuro2a WT neuro2a wild-type, Neuro2a Panx1 KO neuro2a pannexin 1 knockout, Neuro2a Panx1 OE pannexin 1-overexpressing neuro2a, Pbcd probenecid, RA retinoic acid

To probe mechanisms underlying the effects of Panx1 on behavioral allodynia, we first established WT, *Panx1* KO, and *Panx1*-overexpressing Neuro2a cell lines to examine the impact of Panx1 on neuronal morphology and cell excitability (Additional file 1: Fig. S3a). Morphologically, Neuro2a cells extended many more neurites in the WT and *Panx1*-overexpressing cells after 5 d of 40 µmol/L RA treatment compared to the *Panx1*-deleted group (Fig. 2a). RA did not enhance the percentage of neurite-bearing *Panx1*-null Neuro2a cells. The fraction of neurite-bearing Neuro2a cells in the absence of RA from 3 to 5 d was low in both WT and *Panx1*-overexpression groups and much lower in *Panx1* KO cells, but it was substantially higher in both WT and especially in *Panx1*-overexpressing Neuro2a cells after RA treatment (Fig. 2b). To confirm that neurite extension was cell autonomous with regard to *Panx1* expression, *Panx1* deleted Neuro2a cells were transfected with an EGFP-tagged m*Panx1* plasmid. In the same dish, neurite growth was much more elaborate in the EGFP-*Panx1* group with RA treatment at 5 d in striking contrast to the surrounding *Panx1*-KO cells (Additional file 1: Fig. S3b).

Concerning cell excitability, inward currents were more robust in cells overexpressing *Panx1* and markedly weaker in Neuro2a *Panx1* KO cells, compared to WT Neuro2a cells. Following 40 µmol/L RA treatment, inward currents were significantly increased in *Panx1*-overexpressing cells and slightly increased in WT cells, whereas currents were not enhanced by RA in *Panx1* KO cells (Fig. 2c). Furthermore, cells were continuously subjected to the Panx1 blocker Pbcd (1 mmol/L) for 5 d of RA treatment to determine whether these differences depended on functional Panx1 channels. Representative recordings (Fig. 2d) and current density analysis (Fig. 2e) revealed the diminished peak currents in Neuro2a WT cells after blocking Panx1 channels. These results indicated that Panx1 channels enhance both neurogenesis and cell excitability and therefore would be expected to impact neuronal differentiation during development and network remodeling.

### Panx1 impacts DRG neurogenesis via Wnt/β-catenin pathway

**Fig. 3:**
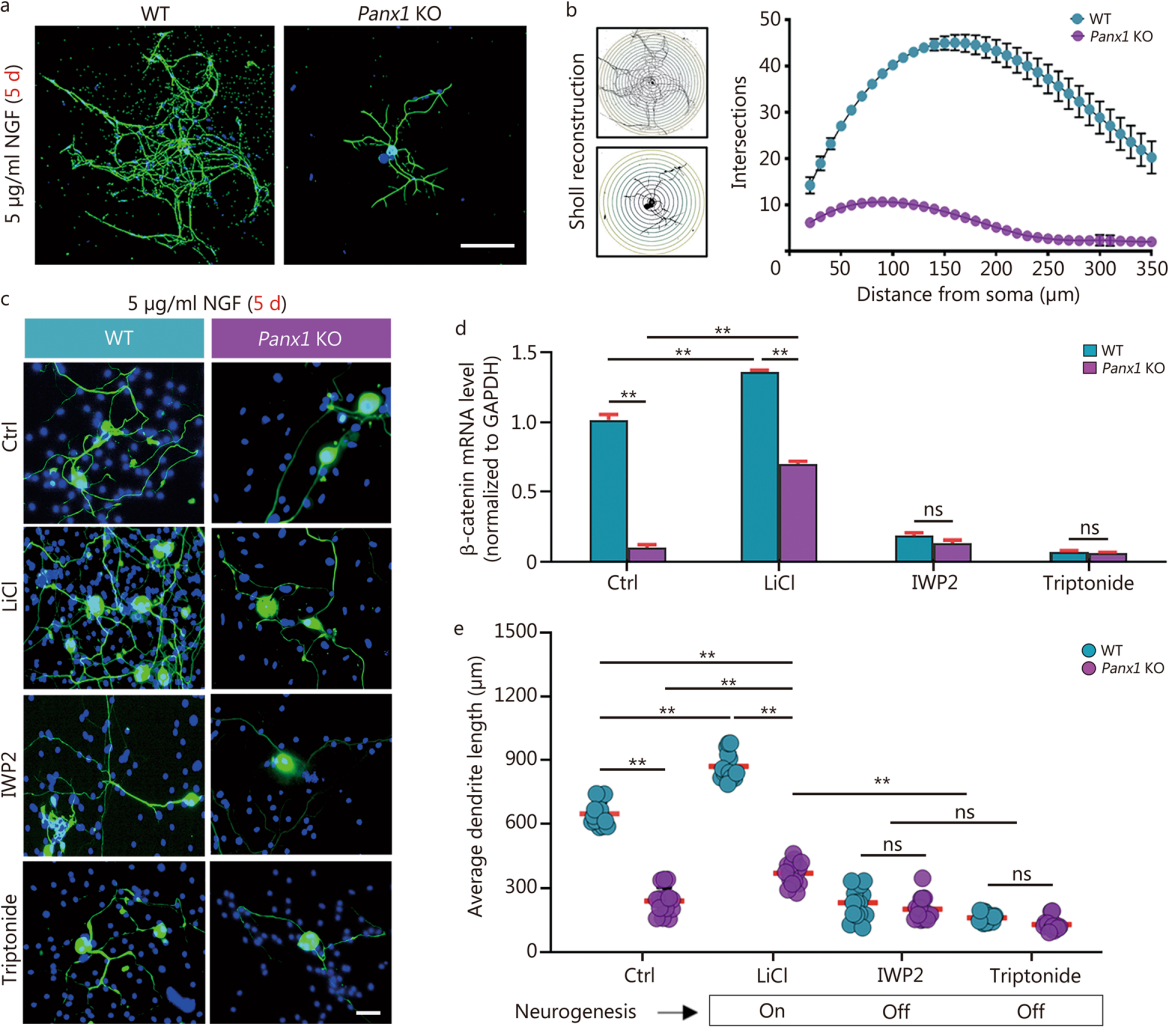
Global *Panx1* deletion impacts DRG neurogenesis via Wnt/β-catenin pathway. **a** cell morphology of NF-200 stained DRG neuron from WT and *Panx1* KO mice after 5 d of NGF treatment. **b** Sholl analysis of intersection numbers in WT and *Panx1* KO neurons (WT vs. *Panx1* KO group, *P* < 0.01). **c** Following 5 d of treatment with NGF, cell morphology of DRG neurons (DRGNs) from WT and *Panx1* KO mice was visualized NF200. Exposure to 20 mmol/L LiCl, 30 nmol/L IWP2, and 100 nmol/L Triptonide was compared. **d** mRNA expression of *β-catenin* in WT and *Panx1* KO DRGNs after 5 d of treatment with NGF. Exposure to LiCl, IWP2, and Triptonide was also compared. **e** Quantification of cell morphology indicated neurite lengths in WT and *Panx1* KO DRGNs following treatment with modifiers of the Wnt/β-catenin pathway (*n =* 15 in each group). Two-way ANOVA with Bonferroni in **d**, **e**, interaction *F* (7, 40) = 2.595 in panel **d**, *F* (3, 16) = 183.1 in panel e. ns non-significant, ^*^P < 0.05, ^**^P < 0.01. Scale bar = 100 μm in panels **a**, and scale bar = 20 μm in panels **c**. DRG dorsal root ganglion, IWP2 Wnt production inhibitor 2, LiCl lithium chloride, NF200 neurofilament 200, NGF nerve growth factor, WT wild-type, Panx1 KO pannexin 1 knockout

Next, primary cultures of dissociated L5 DRG sensory neurons were established from WT and *Panx1* KO mice to probe Panx1’s impacts on neurogenesis and cell excitability. Notably, confocal microscopy imaging of neurofilaments from *Panx1* KO DRGNs showed that the cells extended only short processes after 5 d of 5 µg/ml NGF treatment compared to much longer and more elaborate processes seen in WT DRGNs (Fig. 3a). As shown in Fig. 3b, Sholl analysis results revealed a higher number of dendrite intersections with Sholl rings in WT vs. *Panx1* KO DRGNs, particularly in the distal dendritic arbor, indicating Panx1 enhanced neurogenesis.

Since shRNA knockdown of *Panx1* was reported to reduce mRNA levels of *β-catenin* in human melanoma cells, we further explored whether the Wnt/β-catenin signaling was involved in *Panx1* KO DRGNs. For that, we applied 20 mmol/L LiCl (Wnt/β-catenin pathway agonist), 30 nmol/L IWP2 (Wnt inhibitor), and 100 nmol/L Triptonide (β-catenin blocker) during 5 d of NGF treatment. As shown in NF200-stained neuron images (Fig. 3c), the dendrite growth was affected in opposite ways by Wnt/β-catenin agonists or inhibitors. The qPCR results showed the mRNA levels of *β-catenin* were dramatically down-regulated in *Panx1* KO compared to WT neurons in basal and LiCl treatment conditions (Fig. 3d). Our quantitative analysis revealed that the average dendrite length of WT neurons was approximately four times that of *Panx1* KO neurons (Fig. 3e). After LiCl treatment, dendritic growth was stimulated in both WT and *Panx1* KO DRGNs (Fig. 3e), consistent with Wnt signaling activation. However, inhibition of either Wnt or β-catenin almost markedly eliminated dendritic length augmentation in those two groups when IWP2 or Triptonide was added; in contrast, LiCl partially overcame neurite extension inhibition in *Panx1* KO DRGNs, consistent with the downstream signaling via β-catenin (Fig. 3c, e). Collectively, these results indicated that Panx1 could modulate neurogenesis via the Wnt/β-catenin pathway in sensory neurons.

### Panx1 channels activate DRGNs excitability

**Fig. 4:**
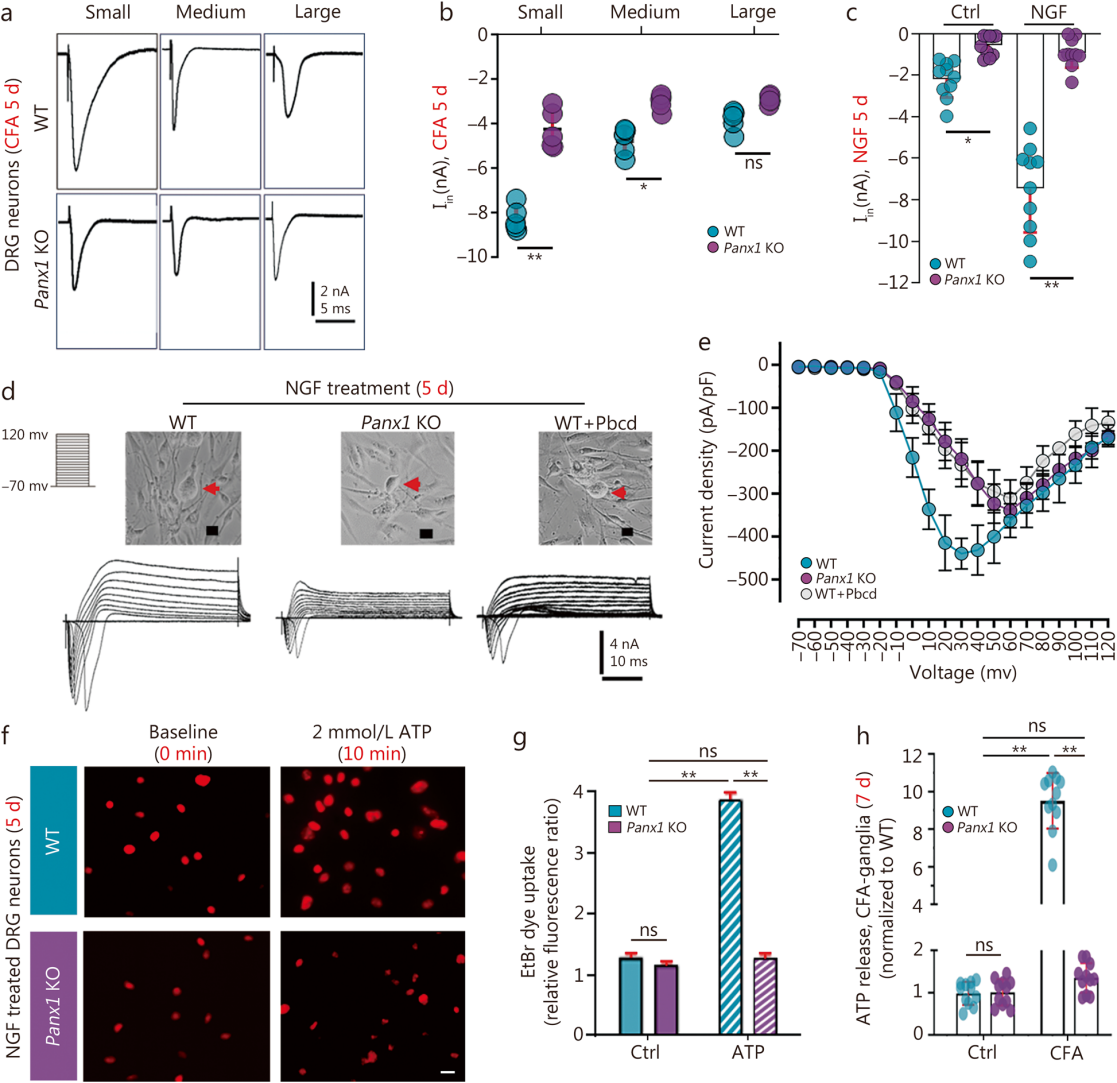
Panx1 enhances NGF-induced inward currents, dye uptake, and ATP release. **a** After 5 d of NGF treatment, representative electrophysiological recordings of different diameter-DRGNs [small (< 20 μm), medium (20–25 μm), and large (> 25 μm)] from WT and *Panx1* KO mice were detected and analyzed (**b**). **c** Maximum inward currents recordings from WT and *Panx1* KO sensory neurons before and 5 d after NGF treatment (Ctrl, 5% serum treated; 5 µg/ml NGF treated; *n =* 10). **d** Representative recordings from WT, *Panx1* KO and Pbcd inhibited Panx1 groups (*n =* 10). **e** Current density analysis of those three types of DRGNs above. **e** Statistical analysis of **c**. **f** EtBr dye uptake images and data analysis **(g)** between WT and *Panx1* KO NGF treated DRGNs with and without 2 mmol/L ATP treatment. **h** ATP release levels analysis between WT and *Panx1* KO DRGNs following CFA injection on day 7. Scale bar = 20 μm in **d**, **f**. ns non-significant, ^*^P < 0.05, ^**^P < 0.01 for one-way ANOVA in **b**, **c**, **g**, **h**. Ctrl control, DRGNs dorsal root ganglion neurons, EtBr ethidium bromide, NGF nerve growth factor, WT wild-type, Panx1 KO pannexin 1 knockout, Pbcd probenecid

Given that pain is transduced by small diameter nociceptors, we assessed the inward currents in DRGNs of various sizes [small (< 20 μm), medium (20–25 μm), and large (> 25 μm)] isolated from ganglia of the CFA treated mice (7d). Representative recordings (Fig. 4a) and quantitative analysis (Fig. 4b) revealed that the amplitudes of the transient inward currents recorded from small DRGNs were significantly lower in *Panx1* KO [(3521 ± 512.2) pico ammeter (pA)] than in the WT group [(6411 ± 651.6) pA] after 7 d of CFA treatment. This excitability difference was consistent with the lower pain sensitivity and allodynia in these animals.

To determine the extent to which Panx1 contributed to the heightened neuronal excitability following CFA treatment, we compared inward currents in DRGNs from WT mice with those lacking *Panx1* or in which Panx1 function was blocked. As shown in Fig. 4c, the amplitude of inward currents recorded from *Panx1* KO DRGNs was smaller than those in WT without CFA treatment, and this difference was markedly enhanced following 5 d in the presence of NGF. Similarly, blocking Panx1 channels by 1 mmol/L Pbcd significantly reduced the inward current amplitude and density of DRGNs (Fig. 4d, e). These results confirmed that functional Panx1 channels greatly augment cellular excitability in DRGNs.

Additionally, we explored the mechanism by which Panx1 channels contribute to pain sensitivity and allodynia. Measurement of the uptake of EtBr dye showed that membrane permeability of WT DRGNs was robustly increased in response to extracellular 2 mmol/L ATP (allogeneic molecule), while *Panx1*-null neurons did not show appreciably increased EtBr uptake in response to ATP application (Fig. 4f, g). Consistent with these dye uptake results, ATP release levels were much higher in WT DRG than in *Panx1* KO ganglia from CFA-treated mice (7 d) (Fig. 4h). These results indicated that ATP-activated Panx1 channels were critical to the up-regulation of pain sensitivity in sensory neurons.

### Panx1 in glia impacts exaggerated neuronal excitability

**Fig. 5:**
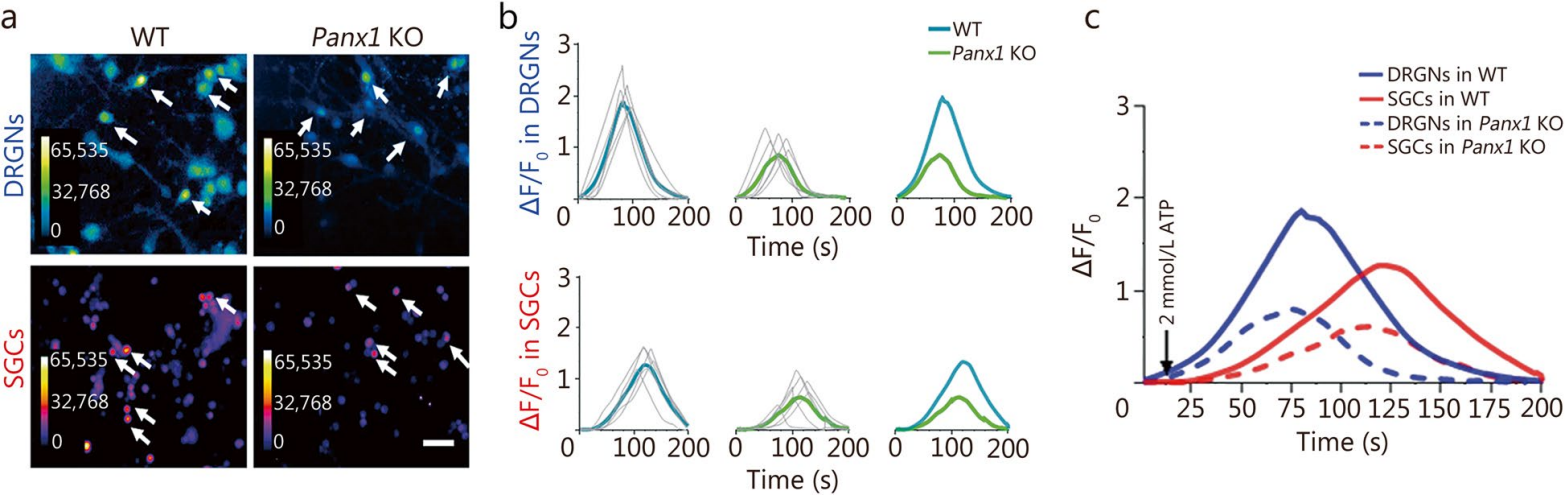
Both dorsal root ganglion neurons (DRGNs) and satellite glial cells (SGCs) show heightened sensitivity to ATP in co-cultures from CFA-treated mice. **a** Ca^2+^ imaging of Fura2-loaded dissociated WT and *Panx1* KO DRGNs and SGCs. Scale bar = 50 μm. **b** Quantification of calcium responses from DRGNs (blue) and SGCs (red) [*n =* 5; statistical analysis from either DRGNs or SGCs indicates that there are stronger calcium waves in WT, compared to *Panx1* KO group (*P* < 0.01)]. **c** Data summarized and replotted from data sets in **b** (downward-pointing arrow indicates a time of ATP application). ns non-significant, ^*^P < 0.05, ^**^P < 0.01 for one-way ANOVA with repeated measures in **c**. WT wild-type, Panx1 KO pannexin 1 knockout

To determine whether ATP sensitivity was different in DRGNs and SGCs in sensory ganglion, intracellular Ca^2+^ changes evoked by ATP were measured in Fura-2 AM-loaded cells dissociated from DRG ganglion after CFA administration to the hindlimb paw (7 d). As shown in Fig. 5a, applying 2 mmol/L ATP enhanced Ca^2+^ responses in DRGNs and the SGCs surrounding them. Further calcium imaging analysis revealed there were smaller and slower Ca^2+^ rises in SGCs than in neurons (Fig. 5b, c). Notably, Ca^2+^ responses in both cell types were lower in *Panx1* KO than in WT cultures (Fig. 5b). This lower ATP sensitivity in SGCs than in DRGNs from *Panx1* KO mice indicates that Panx1 in glia also impacts exaggerated neuronal excitability, although its impact is less than in neurons.

### Neuronal Panx1 drives neurogenesis and hyperexcitability in DRG following CFA injection in paw

**Fig. 6:**
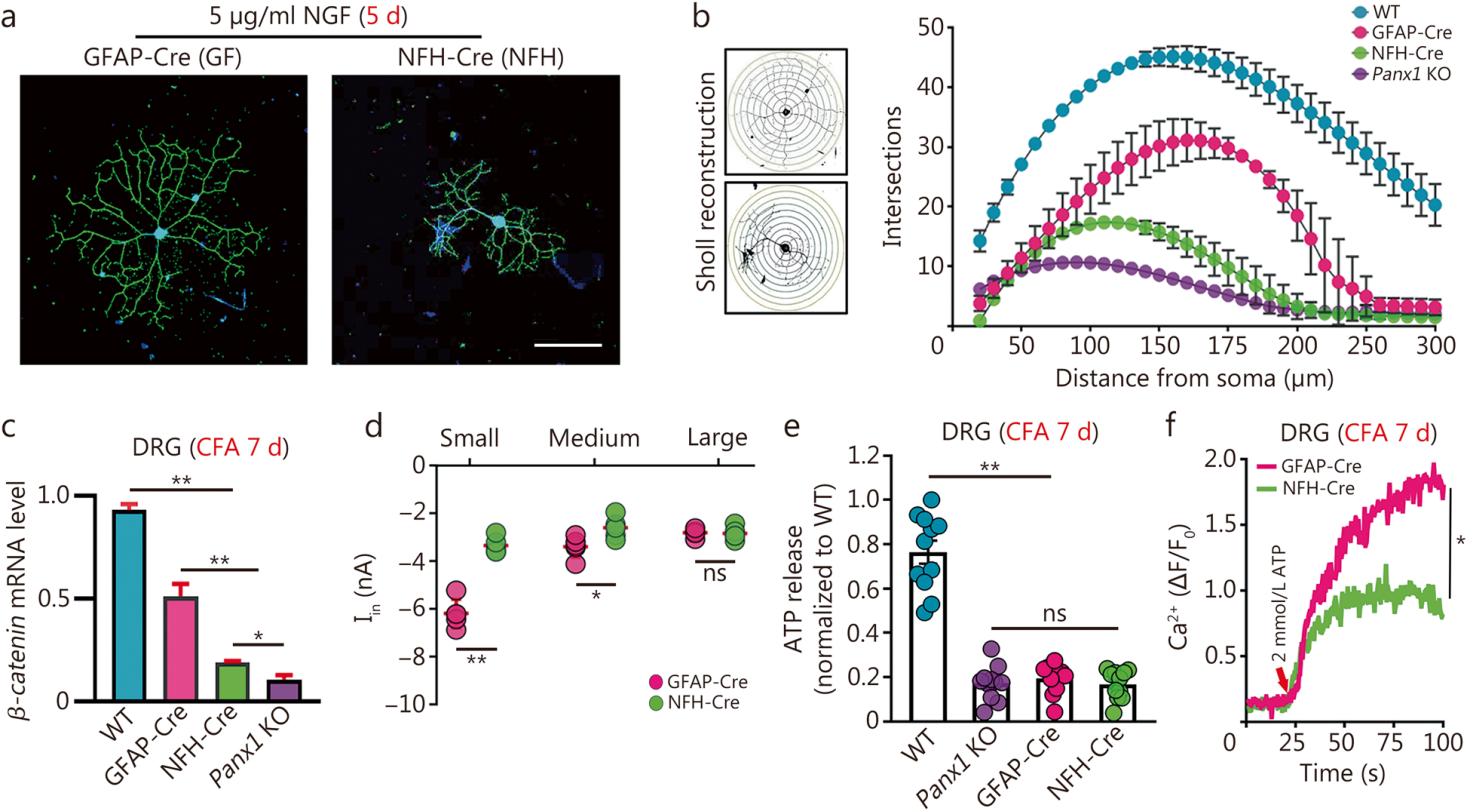
*Panx1* contributes to DRG changes in both neurons and glia in chronic inflammatory pain. **a** Representative morphologies of dorsal root ganglion neurons (DRGNs) from GFAP-Cre and NFH-Cre mice stained with NF200 antibody (green) after 5 d of NGF induction. Scale bar = 100 μm. **b** Sholl analysis of NGF-treated GFAP-Cre and NFH-Cre DRGNs on day 5. **c***β-catenin* mRNA analysis of those NGF-treated GFAP-Cre and NFH-Cre DRGNs compared to WT and global *Panx1* KO [*n =* 6; NFH-Cre vs. GFAP-Cre group (*P* < 0.01), global *Panx1* KO vs. NFH-Cre group (*P* < 0.05), global *Panx1* KO vs. GFAP-Cre group (*P* < 0.01), WT vs. NFH-Cre or GFAP-Cre or global *Panx1* KO group (*P* < 0.01)]. **d** Quantification of inward currents from GFAP-Cre and NFH-Cre DRGNs (traced with DiI) projecting to CFA-injected hind paw on day 7 (*n =* 8). **e** ATP release analysis of WT, *Panx1* KO, GFAP-Cre and NFH-Cre DRG ganglia 7 d post-CFA injection (*n* = 10). **f** Ca^2+^ response analysis of GFAP-Cre and NFH-Cre DRGNs after CFA injection on day 7 (*n =* 5). ns non-significant, ^*^P < 0.05, ^**^P < 0.01 for one-way ANOVA in **b, c, d, e, f** with repeated measures. CFA complete Freund’s adjuvant, Panx1 pannexin 1, Panx1 KO pannexin 1 knockout, DRG dorsal root ganglion, GFAP-Cre glial fibrillary acidic protein-Cre, NFH-Cre neuro filament H-Cre, NF200 neurofilament 200, NGF nerve growth factor, WT wild-type

To probe the cell-specific role of Panx1 in DRG following CFA injection, a detailed analysis of dendrite development, cell excitability, and Ca^2+^ influx was performed in neurons from mice with targeted Panx1 deletion in neurons (NFH-Cre) and glia (GFAP-Cre). Sholl analysis results showed that neurotic arbors were more complex in both NFH-Cre and GFAP-Cre groups compared to global *Panx1* KO, with neuron-targeted *Panx1* deletion leading to a more severe reduction in intersection number than in GFAP-targeted *Panx1* deletion (Fig. 6a, b). Further analysis of dendritic complexity in all genotypes indicated that a lower secondary dendrite length in NFH-Cre prominently affected the total branch length of DRGNs, even though there was no difference in branch numbers and primary dendrite length between GFAP-Cre and NFH-Cre groups (Additional file 1: Fig. S4a, S4b). Interestingly, the *β-catenin* mRNA level in GFAP-Cre was higher than in the NFH-Cre group, indicating that neuronal Panx1 contributes to neurogenesis (Fig. 6c). As shown in Fig. 6d, the inward current amplitude of GFAP-Cre mice was remarkably higher in smaller DRGNs compared to NFH-Cre mice, implying that neuronal *Panx1* deletion evoked greater excitability of the small peripheral nociceptive cells than deletion in glia. Moreover, all *Panx1*-deleted mice showed lower ATP levels in DRG, but differences among the genotypes were not detected (Fig. 6e). However, intracellular Ca^2+^ responses of dissociated DRGNs were considerably higher in the GFAP-Cre than in the NFH-Cre group after 7 d post-CFA injection (Fig. 6f). Collectively, these results indicated that neuronal Panx1 contributes substantially to hyperexcitability following CFA injection.

The more prominent role of neuronal Panx1 in paw sensitization than in orofacial pain might be due to differential expression in ganglia innervating the sites of inflammation. To test for this, we used staining for β-gal to assess *Panx1* levels in sensory ganglia from mice in which glial or neuronal Panx1 was deleted. Results showed the absence of staining in TG and DRG from WT mice, which lack the β-gal transcript; however, in ganglia of both *Panx1*-nulls, β-gal staining was prominent in brightfield images (Additional file 1: Fig. S5a). SGC staining for glutamine synthetase aided in identifying sensory neurons that they encircled in the merged images (Additional file 1: Fig. S5a). Quantification of nine DRG and TG sections from three mice revealed significantly higher expression of the *Panx1* reporter gene in the DRG than in the TG (Additional file 1: Fig. S5b) and significantly higher non-neuronal β-gal staining in the TG than in the DRG (Additional file 1: Fig. S5c). These results indicate that cellular expression levels of Panx1 are different in the TG and the DRG, with Panx1 being more prominent in DRGNs and Panx1 more prominent in TG glia. This difference likely contributes to the higher relevance of SGC Panx1 to orofacial pain and the greater contribution of neuronal Panx1 to hindlimb inflammatory hypersensitivity.

## Discussion

Panx1 contributes to many physiological and pathological processes, such as migraine headaches [45], spinal cord injury [33], sciatic nerve injury [32], cancer pain [46, 47], and inflammatory pain [48]. For example, blockade of Panx1 activation reduced cortical susceptibility to cortical spreading depression in migraine aura pathogenesis [45]; Panx1 blockers (10Panx peptide, carbenoxolone, and Pbcd) decreased mechanical hyperalgesia by inhibiting nocioceptor ion channels [NMDA receptors (NMDAR) and P2X7 purinoceptors (P2X7R)] in a spared nerve injury rat model [33]. These studies provide strong evidence that the Panx1 channel may provide a key element in the process of pain sensitization, both in the brain and in the periphery. The present study revealed that global/neuron-*Panx1* deletion in mice rapidly increased pain thresholds in an experimental plantar inflammatory pain. These findings confirmed our previous report that *Panx1* deletion provided substantial protection against the development and maintenance of allodynia in an orofacial inflammatory mouse model [17]. In an initial study on rats following sciatic nerve ligation [18], *Panx1* was increased in DRG but not in the spinal cord dorsal horn during neuropathic pain development, implying that the locus of sensitization was within the DRG [21, 49]. Our investigation demonstrated that gene expression of *Panx1* was dramatically elevated in DRG but not in the spinal cord from 7 to 21 d after CFA injection. Therefore, because cells in the DRG are accessible to drugs delivered systemically, there is enormous potential value in developing therapeutics that target the Panx1 channel in treating inflammatory pain.

Neurite regeneration has a strong correlation with pain sensitization, which is critical for alleviating neuropathic pain [50]. Concerning the role of neurite outgrowth in painful conditions, it is noteworthy that injury induces local branching of nociceptors in the skin [51]. Our study has revealed that neurite extension is retarded when Panx1 expression or function is blocked. However, these results are in contrast with those of other studies that examined the effects of Panx1 deletion or blockade on neurite extension. Prolonged (36 h) probenecid and *Panx1* siRNA treatment led to increased neurite numbers in Neuro2a cells and ventricular zone neural progenitors, while transfection of neural progenitors with GFP-tagged *Panx1* led to fewer cells with neurites [52]. The hypothesized mechanism for the negative impact of Panx1 on neurite expression involved microtubule cytoskeleton stabilization [53]. A recent study showing higher activity of dendritic protrusions in developing cortical neurons from *Panx1*-null mice (with rescue by *Panx1* expression) was similarly interpreted as indicating increased process stability due to Panx1 binding to cytoskeleton [54]. One possible explanation for different results obtained from *Panx1*-null mice is that previous publications used transgenic mice whose coding region was only partially deleted and with a genetic background prone to passenger mutations. This effect has been demonstrated to result in the deletion of *Caspase-4* and several other gene mutations in this transgenic *Panx1* mouse [55]. In contrast, *Panx1*-null mice used in the present study were generated with the C57BL6 background by crossing mice obtained from the KOMP project [21]. These mice are hypomorphs, expressing deficient mRNA levels and having virtually no Panx1 function.

The changes that we observed in pain sensitivity and neuronal excitability in mice lacking functional Panx1 presumably result from altered signaling pathways linked to Panx1 function. One such pathway is Wnt signaling, where recent study has revealed that pathway components, including Wnt-3a, Frizzled 4, and β-catenin, rapidly increased at both mRNA and protein levels in rat L4–6 DRG in neuropathic pain [56]. Notably, *β-catenin* levels were found to be lower in periosteal bone from *Panx1* KO mice compared to WT, indicating that mechanoresponsive Wnt signaling was impaired in *Panx1*-null osteocytes [57], and *β-catenin* expression declined when *Panx1* was knocked down in melanoma cells [58, 59]. Consistent with these findings, our results confirmed that the *β-catenin* level was lower in the *Panx1* KO DRGNs, compared to WT mice. Given the critical role of Wnt/β-catenin pathway activation in mediating neuronal-related gene expression, cell survival, and cytoskeletal organization [60–62], we tested *β-catenin* expression using the Wnt signaling agonist (LiCl) and inhibitors (IWP2, Triptonide) on WT and *Panx1-*null DRGNs. These studies revealed that Panx1 could modulate neurogenesis via activating the Wnt/β-catenin pathway in DRGNs, suggesting that this pathway may represent the downstream effector of neurogenesis changes. Thus, it can be speculated that Panx1 interacts with Wnt signals to enhance pain intensity.

Allodynia depends in part on peripheral sensitization, which involves neuronal hyperexcitability. It is well known that Neuro2a/DRG cells extend neurites and display inward Na^+^/Ca^2+^ currents in response to treatment with the differentiating agent RA and NGF [18, 35, 36, 63–65]. Our results demonstrated that, in both RA-treated Neuro2a cells and NGF-treated DRGNs, Panx1 was required for neurite extension and robust expression of cellular excitability. ATP, an allogeneic molecule, is released through Panx1 channels [58, 66], which might also contribute to pain sensitivity and allodynia. ATP release from cultured neurons and extracted DRGs was significantly enhanced in the hind paw CFA-induced inflammatory pain model, and this enhancement was absent in *Panx1*-null DRG and neurons cultured therefrom. Additionally, ATP-elicited Ca^2+^ elevations were blunted in both neurons and glia from *Panx1*-null mice, suggesting that ATP signaling might contribute to the phenotypic differences in *Panx1*-null and WT mice. These results are similar to those obtained in imaging studies of TG, in which submandibular CFA led to heightened Ca^2+^ responses to both K^+^ and ATP applications compared to controls [17]. These data indicated that Panx1 channels activate DRG neuron excitability via ATP.

The excitability of primary sensory neurons is pathologically enhanced following tissue injury and inflammation [67]. Recent investigations have indicated that pharmacological inhibition or genetic deletion of Panx1 strongly attenuated the excitability of CA1 pyramidal neurons [68] and ATP release in hippocampal slices [69], suggesting that Panx1 might also reduce the nociceptive threshold by affecting the transmission and persistence of such excitability in peripheral neuralgia. The present study data showed that *Panx1* deletion reduced basal inward currents in both Neuro2a cells and DRGNs during neuronal differentiation, while current densities also decreased after Panx1 blockade by 1 mmol/L Pbcd. While supportive of the deletion study, Pbcd is an organic anion transport inhibitor that not only inhibits Panx1 but also affects other cell functions [70]. Generally, smaller diameter DRG cells are nociceptors and thermoreceptors, and larger high threshold mechanoreceptors become recruited into activity in pain sensitization. In this study, it was also observed that electrophysiology of small-diameter DRGNs was improved after *Panx1* deletion, indicating that the Panx1 channels particularly impacted nociceptive C- and Aβ-fiber cells that mediate pain sensitization [33, 35, 65].

Our initial behavioral studies showed that pain thresholds were higher in global *Panx1*-deleted than in WT mice and that neuron-specific deletion also increased pain threshold, albeit not quite as effectively. Because we previously showed that glial-specific deletion effectively raised the pain threshold in an orofacial model [17], it was hypothesized that glial-targeted *Panx1* deletion would provide at least partial relief from hypersensitivity. However, it did not significantly raise the threshold in the hind paw inflammatory pain model compared to that in WT mice. Given ATP release of DRGNs was much higher in WT than in *Panx1* KO after CFA injection, we further compared neuron and glial sensitivity to ATP. Measurement of Ca^2+^ signaling in SGCs or neurons revealed that both cell types were hyperactive. Intracellular Ca^2+^ elevation evoked by ATP in DRGNs was higher and more rapid than in SGCs; however, both were diminished in *Panx1*-null cells. These findings indicate that reduced participation of glial cells in inflammatory signaling might also contribute to the lesser behavioral hypersensitivity in the knockout mice. Therefore, pain sensitivity has a close association with cell-specific *Panx1* in DRG.

Further dendrite growth and cell excitability analysis supported the view that both neuronal and glial Panx1 might contribute to behavioral hypersensitivity in DRG following CFA treatment. On one hand, *Panx1* deletion in glia reduced *β-catenin* expression, and this directly limited Wnt/β-catenin signaling induced neurite remodeling [40, 62, 71], resulting in a decrease in dendritic extension. On the other hand, neuronal *Panx1* deletion evoked greater excitability of the small peripheral nociceptive cells than deletion in glia. These interesting data contrast with the striking efficacy of glial Panx1 in TG accompanying inflammatory orofacial pain [17], and indicate that the neuronal Panx1 is more prominent in controlling neurogenesis and hyperexcitability in the DRG than in the TG in inflammatory pain.

This difference likely arises from distinct properties of the neurons that innervate the hind paw and submandibular skin. The sensory neurons innervating these areas are located in the DRG and TG, respectively. Gene expression comparisons have revealed that significant differences in profiles, including a study comparing both transcriptomes of the ganglia and translatomes from Scn10a-positive nociceptors in both DRG and TG [34]. In that study, the overall level of *Panx1* transcript was 2.6-fold higher in the DRG than in the TG, whereas translated mRNA was > 5-fold higher in the DRG than in TG nociceptors. Our demonstration of more abundant β-gal reporter activity in DRGNs than in TG neurons is consistent with these findings. Moreover, the higher β-gal reporter activity in non-neuronal cells in TG provides additional support for differential cellular involvement of Panx1 in these ganglia [34]. Although we found that neuronal Panx1 played a prominent role in controlling pain sensitization in inflammatory pain, the mechanisms underlying how neuronal Panx1 impacts synapse formation and nociceptive ion channels are still not known and need to be further studied. Collectively, our results revealed that the prominent role of neuronal Panx1 in neurogenesis and excitability following hindlimb CFA injection may provide a novel target for intervention in pain management.

Despite the dramatically lower pain sensitivity in mice lacking neuronal Panx1 in our study, interpretation is limited because the mechanistic analyses reported here were only performed in cell culture. Future studies should examine whether Panx1-related differences in neurogenesis exist in both DRG and receptive fields within the skin. Our findings also predict that Panx1-targeted therapy might benefit inflammatory pain, and future studies should optimize such pharmaceutical compounds.

## Conclusions

The present study demonstrated that neuronal Panx1 is crucial for initiating and maintaining pain hypersensitivity following hind paw inflammatory injury. Mechanisms by which Panx1 facilitates pain sensitivity include enhanced neurite outgrowth, exaggerated cell excitability, and amplified ATP-induced calcium influx. Signaling via the Wnt/β-catenin pathway appeared to underlie Panx1 actions. In conclusion, the present study indicated that Panx1 is a prominent “pain driver” in peripheral hypersensitivity via cell-autonomous effects on neuronal excitability.

### Supplementary Information

**Additional file 1: Table S1** Primer pairs for qRT-PCR. **Fig. S1** Detailed pain threshold analysis in WT, global Panx1 KO, NFH-Cre, and GFAP-Cre Panx1 KO mice after CFA inflammation. **Fig. S2** Pain threshold baseline studies on all genotypes before CFA injection. **Fig. S3** Neural differentiation studies on Panx1 gene-modified Neuro2a cells. **Fig. S4** Detailed Sholl analysis on DRGNs from Panx1-deleted genotypes. **Fig. S5** β-gal staining in DRG and TG tissue sections.

## Data Availability

The data used to support the findings of this study are available from the corresponding authors upon reasonable request.

## Abbreviations

ATP: Adenosine triphosphate
β-gal: β-galactosidase
CFA: Complete Freund’s adjuvant
DAPI: 4’6-Diamidino-2-phenylindole
DRGNs: Dorsal root ganglion neurons
EGFP: Enhanced green fluorescent protein
EtBr: Ethidium bromide
GFAP-Cre: Glia-specific Panx1 KO mice (GFAP-Cre: Panx1^f/f^)
NF200: Neurofilament 200
NFH-Cre: Neural-specific Panx1 KO mice (NFH-Cre: Panx1^f/f^)
NGF: Nerve growth factor
Panx1 KO: Pannexin1 knock-out
Panx1: Pannexin1
pA: pico ammeter
Pbcd: Probenecid
RA: Retinoic acid
SGCs: Satellite glial cells
TG: Trigeminal ganglia
WT: Wild-type
