# Supplementary material for: Neuronal Panx1 drives peripheral sensitization in experimental plantar inflammatory pain
Source: Mil Med Res. 2024 Apr 29;11:27. doi: 10.1186/s40779-024-00525-8 (PMC11057180; doi:10.1186/s40779-024-00525-8)
Supplement: Supplementary file 1 — Additional file 1: Table S1 Primer pairs for qRT-PCR. Fig. S1 Detailed pain threshold analysis in WT, global Panx1 KO, NFH-Cre, and GFAP-Cre Panx1 KO mice after CFA inflammation. Fig. S2 Pain threshold baseline studies on all genotypes before CFA injection. Fig. S3 Neural differentiation studies on Panx1 gene-modified Neuro2a cells. Fig. S4 Detailed Sholl analysis on DRGNs from Panx1-deleted genotypes. Fig. S5 β-gal staining in DRG and TG tissue sections. [file 40779_2024_525_MOESM1_ESM.pdf]

Table S1 Primer pairs for qRT-PCR

Gene ID	Symbol	Accession#	Primer sequence (5'-3') [a]	Base pairs [b]
55991	*Panx1*	NM_019482.2	F: CAGGCTGCCTTTGTGGATTC	-
			R: CGGGCAGGTACAGGAGTATG	145
406218	*Panx2*	NM_001002005.2	F:GGTACCAAGAAGGCCAAGACT	159
			R: GGGGTACGGGATTTCCTTCTC	
208098	*Panx3*	NM_172454.2	F: CTTACAACCGTTCCATCCGC	-
			R: CAGGTACCGCTCTAGCAAGG	140
14609	*Cx43*	NM_010288.3	F: ACAGCGGTTGAGTCAGCTTG	-
			R:GAGAGATGGGGAAGGACTTGT	106
546729	*Calhm1*	NM_001081271.1	F: CTGCTGACCACATTACTAGCG	-
			R:CTGTGCATGTCTCATCGAAGG	133
12387	*β-catenin*	NM_007614.3	F: TGCTGAAGGTGCTGTCTGTC	-
			R: CTGCTTAGTCGCTGCATCTG	158
14433	*GAPDH*	NM_001290631.1	F: TGGATTTGGACGCATTGGTC	-
			R: TTTGCACTGGTACGTGTTGAT	211

Primer pairs for quantitative Real Time PCR, [a]Primer direction (F-forward; R-reverse) and [b]amplification products size in base pairs, - no data.

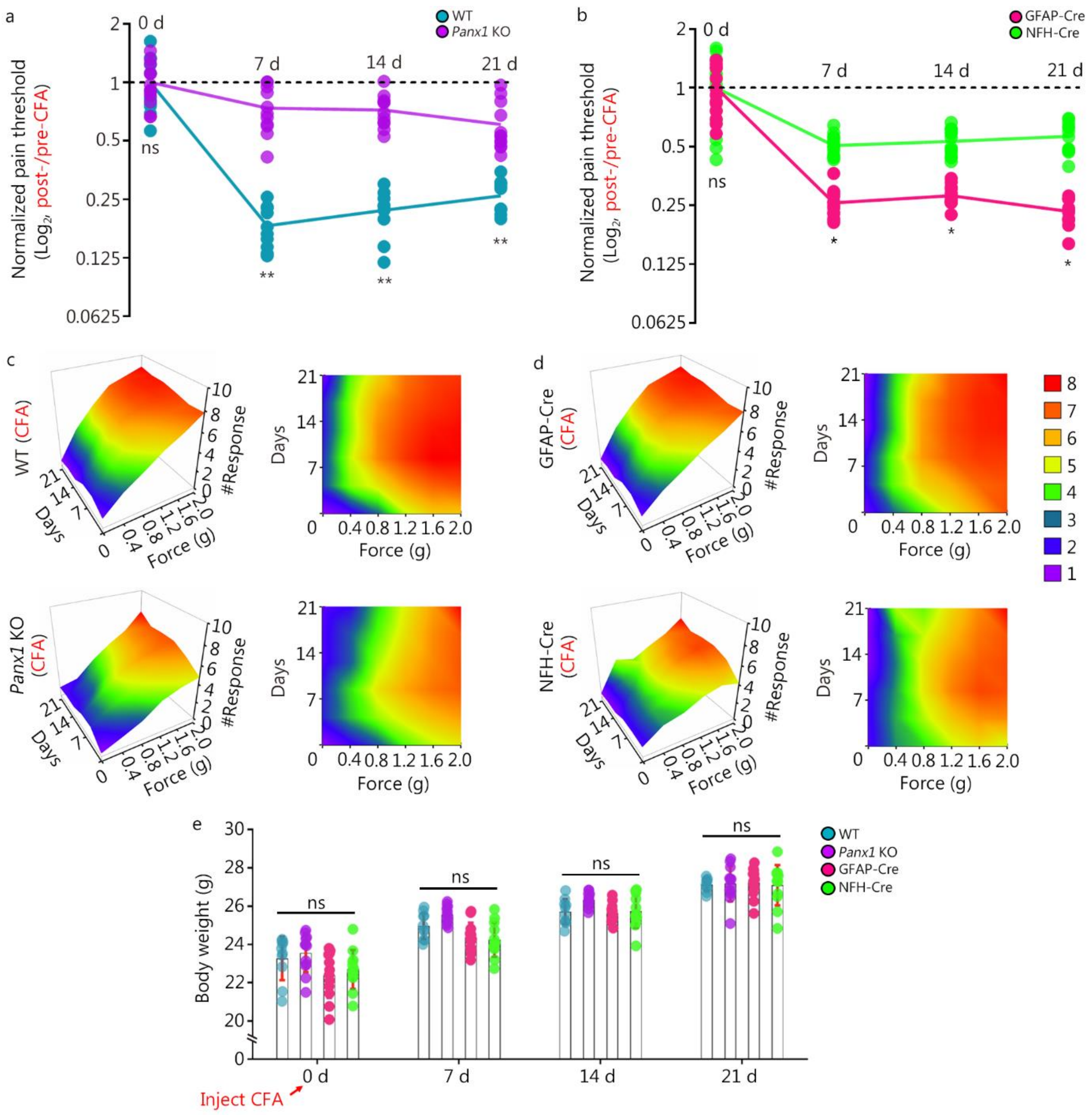

Fig. S1 Detailed pain threshold analysis in WT, global Panx1 KO, NFH-Cre, and GFAP-Cre Panx1 KO mice after CFA inflammation. **a** Normalized pain threshold assessment (post-/pre-CFA injection) in WT and *Panx1* KO mice. **b** Normalized Pain threshold assessment (post-/pre-CFA) in NFH-Cre and GFAP-Cre *Panx1* KO mice. **c** Topological assessment (3D and 2D heatmaps) in WT and *Panx1* KO mice after CFA injection. Scale bar = 20 μm. **d** Topological assessment (3D and 2D heatmaps) in NFH-Cre and GFAP-Cre *Panx1* KO mice after CFA injection. Scale bar = 20 μm. **e** Dynamic comparison of body weight in the four mouse genotypes above following CFA injection. n = 11 in WT group, n = 11 in *Panx1* KO group, n = 12 in GFAP-Cre group, and n = 12 NFH-Cre group. Two-way ANOVA with Bonferroni

correction in **a**, **b**, and **e**, interaction F (3, 82) = 10.16 in panel **a**, F (3, 100) = 4.262 in panel **b**, F (9, 144) = 1.787 in panel **e**. ns non-significant, $^{*}P < 0.05$, $^{**}P < 0.01$. CFA complete Freund's adjuvant, DRG dorsal root ganglion, NFH-Cre neuro filament H-Cre, GFAP-Cre glial fibrillary acidic protein-Cre, Panx1 pannexin 1, Panx1 KO pannexin 1 knockout, WT wild-type

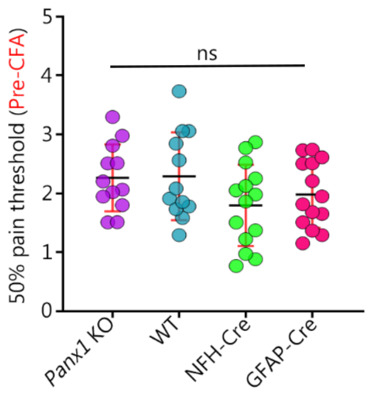

Fig. S2 Pain threshold baseline studies on all genotypes before CFA injection. There was no significant difference in 50% pain threshold baselines in WT, Panx1 KO, NFH-Cre, and GFAP-Cre Panx1 KO mice DRG neurons before CFA injection. n = 11 in WT group and *Panx1* KO group, n = 12 in NFH-Cre group and GFAP-Cre. ns non-significant. CFA complete Freund's adjuvant, DRG dorsal root ganglion, NFH-Cre neuro filament H-Cre, GFAP-Cre glial fibrillary acidic protein-Cre, Panx1 pannexin 1, Panx1 KO pannexin 1 knockout, WT wild-type

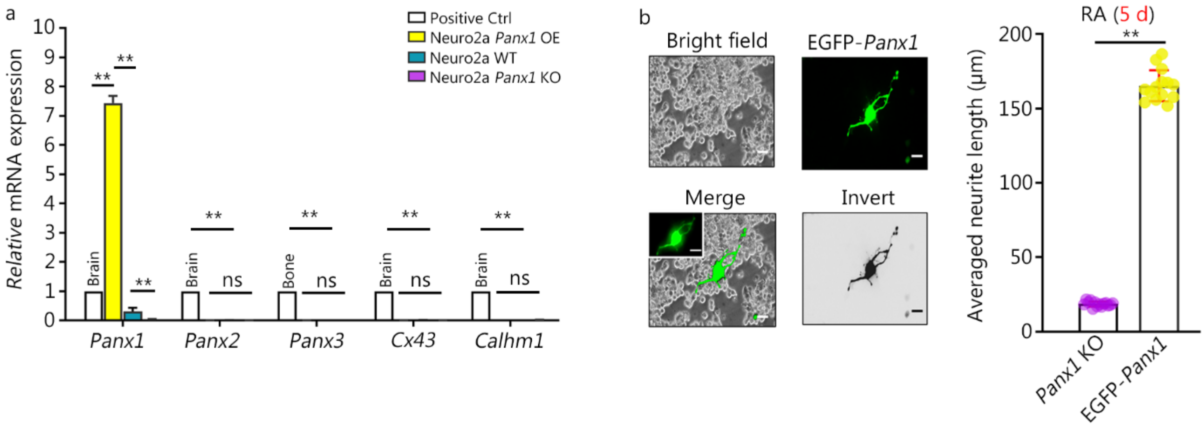

Fig. S3 Neural differentiation studies on Panx1 gene-modified Neuro2a cells. **a** Characterization of expression levels of mRNA encoding several large ion channels in the Neuro2a cell lines used in these studies: Neuro2a WT, Neuro2a Panx1 KO, and Neuro2a *Panx1* OE cells. All expression data are normalized to brain sample, except for Panx3, for which bone was used as a positive control because Panx3 is not expressed in brain tissue. Note that Panx1 was present at low levels in parental Nero2a cells and was highly overexpressed in the transfected cells, whereas other large channels tested were not present in parental Neuro2a cells or induced in transfectants (*Panx2*, *Panx3*, *Cx43*, *Calhm1*). **b** Neurite extension studies on co-cultures of *Panx1* KO Neuro2a cells with a few cells transfected with EGFP-*Panx1* revealed cell autologous enhancement of neurite extension (n = 14). Scale bar = 20 μm in panels **b**. ns non-significant, $^{*}P < 0.05$, $^{**}P < 0.01$ for one-way ANOVA in panel **a**. For panel **b**, $^{**}P < 0.01$ for the paired t-test. Ctrl control, Cx43 connexin 43, Calhm1 calcium homeostasis modulator 1, EGFP enhanced green fluorescent protein, Neuro2a WT neuro2a wild-type, Neuro2a Panx1 KO neuro2a pannexin 1 knockout, Neuro2a Panx1 OE pannexin 1-overexpressing neuro2a, Panx1 pannexin 1, Panx2 pannexin 2, Panx3 pannexin 3, RA retinoic acid

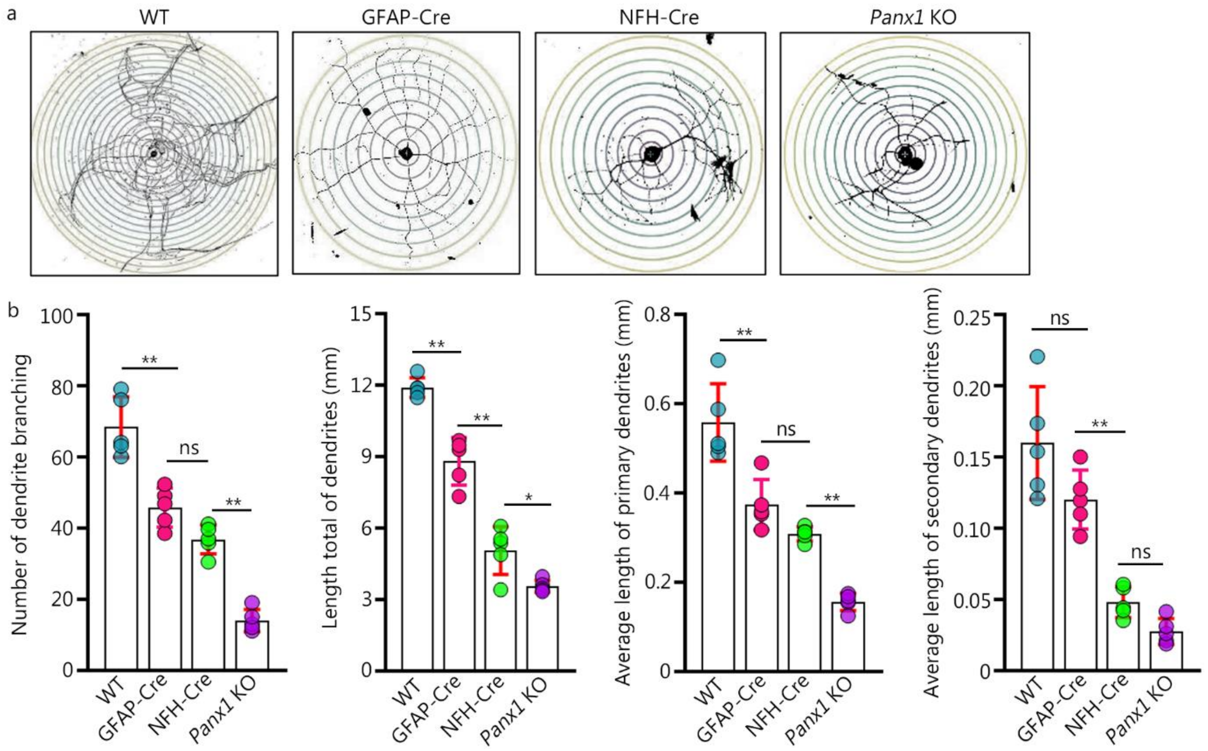

Fig. S4 Detailed Sholl analysis on DRGNs from Panx1-deleted genotypes. **a** Sholl analysis in WT, GFAP-Cre, NFH-Cre, and *Panx1* KO mice DRGNs. **b** Analysis of the number of branches, total length, and average values of primary and secondary dendrites in those four genotypes of DRGNs above. ns non-significant, $^{*}P < 0.05$, $^{**}P < 0.01$ for one-way ANOVA in **b**. DRGNs dorsal root ganglion neurons, GFAP-Cre glial fibrillary acidic protein-Cre, NFH-Cre neuro filament H-Cre, Panx1 pannexin 1, Panx1 KO pannexin 1 knockout, WT wild-type

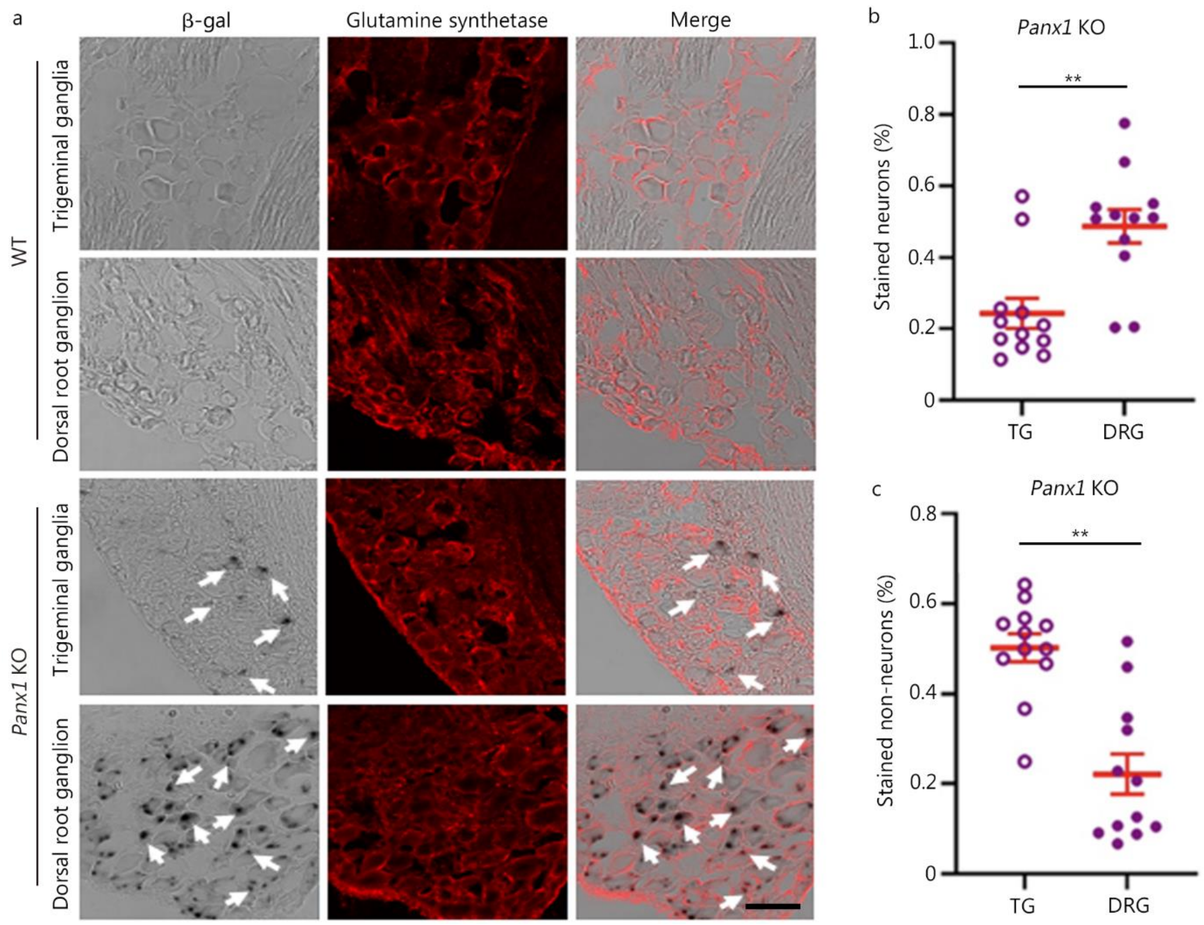

Fig. S5 β-gal staining in DRG and TG tissue sections. **a** Representative brightfield images of β-gal staining, glutamine synthetase (red) and merged on sections from WT or *Panx1* KO mice in TG and DRG, respectively. Histograms showing the mean ± SEM values of the number of β-gal-stained neurons (**b**) and non-neuronal cells (**c**) in TG and DRG, respectively, expressed as a percentage relative to that of the total number of neurons in *Panx1 KO* TG and DRG. Note that β-gal positive neurons were higher in DRG than in TG, but, on the contrary, the number of β-gal positive non-neuronal cells was higher in TG compared to DRG. Scale bar = 50μm in **a**. $^{**}P < 0.01$ for the unpaired Student's *t*-test. Number of β-gal-positive cells was counted on 9 tissue sections per mouse ganglion. β-gal β-galactosidase, DRG dorsal root ganglion, TG trigeminal ganglia, Panx1 pannexin 1, Panx1 KO pannexin 1 knockout, WT wild-type,
